# Influence of Light Irradiation on the Degradation of Dezocine in Injections

**DOI:** 10.3390/pharmaceutics16070858

**Published:** 2024-06-25

**Authors:** Li Zhu, Xu Teng, Yu Duan, Xia Zhang, Jingxin Xie, Mingzhe Xu, Lihui Yin

**Affiliations:** 1 Key Laboratory for Quality Research and Evaluation of Chemical Drugs, National Institutes for Food and Drug Control, Beijing 100029, China; zhuli@nifdc.org.cn (L.Z.); yduane@163.com (Y.D.); s791657998@126.com (X.Z.); xjejx@nifdc.org.cn (J.X.); xumzhe@nifdc.org.cn (M.X.); 2 Department of Laboratory Medicine, Affiliated Qingyuan Hospital of Guangzhou Medical University, Qingyuan People’s Hospital, Qingyuan 511518, China

**Keywords:** dezocine, photodegradation, generation mechanism, degradation efficiency, safety of drug therapy

## Abstract

Dezocine, which is well-known as an analgesic, had about 45% share of the Chinese opioid analgesic market. Since drug products containing impurities could bring serious health consequences, it was important to control the generation of impurities and degradation products in the dezocine product. In this study, two kinds of photodegradation products (i.e., degradation product 1 and degradation product 2) in the dezocine injection were isolated using high-performance liquid chromatography. The possible structures of the photodegradation products were identified using both high-resolution mass spectrometry and nuclear magnetic resonance spectroscopy. In addition, the possible generation mechanism showed that degradation product 1 was the oxidation product of dezocine, and degradation product 2 was the coupled dimer of dezocine. Finally, we found that the degradation rate of dezocine increased with the increase in light intensity. Moreover, the degradation of dezocine easily occurred under ultraviolet light in comparison with visible light. A deeper insight into the generation of the photodegradation products in the dezocine injection would directly contribute to the safety of drug therapy based on the dezocine injection by minimizing the degradant/impurity-related adverse effects of drug preparations.

## 1. Introduction

In 2006, the key principle of the International Conference on Harmonisation (ICH) guidelines was established for evaluating the safety of impurities in pharmaceutical drug substances (ICH Q3A) and drug products (ICH Q3B) [1,2]. Organic impurities that were generated during the synthesis of the drug substance were categorized as synthetic impurities, as defined in ICH Q3A [3,4]. On the other hand, organic impurities that formed during the manufacturing of the drug product or storage were classified as degradation products according to ICH Q3B [5]. During the initial phases of drug development, impurities were deemed safe as long as they did not surpass levels observed in preclinical toxicological safety studies [6]. However, in later stages, impurities were recognized as critical quality attributes according to the ICH guideline and had to be identified if they exceeded the identification thresholds defined by ICH. In the above guidelines, specification limits for impurities and degradation products were established and applied during the release of both the drug substance and drug product [7,8]. As a result, controlling drug impurities became a key focus in pharmaceutical research, encompassing the entire lifecycle of drug products from development and production to approval processes. [9,10]. Moreover, impurities may also be introduced during the storage and transportation processes [11,12]. Therefore, it is essential to characterize, manage, and monitor impurities formed during the storage and transportation processes to ensure drug quality.

Dezocine is a synthetic opioid drug that provides mixed action as a partial µ-receptor agonist and κ-receptor antagonist [13,14]. It was first introduced in the 1970s as an analgesic by Wyeth, which was a United States pharmaceutical company and marketed in the United States [15]. Subsequently, dezocine injection was developed by Yangtze River Pharmaceutical Group in 2009 and has been used as a prescription drug for the relief of postoperative, visceral, and cancer pain. Because of its stronger analgesic effect and lower incidence rate of adverse reactions compared with morphine, it has become the favored opioid analgesic in China. In 2016, dezocine sales in China exceeded USD 630 million, capturing over 45% of the market share. An increasing number of research laboratories have reinitiated the study of dezocine [16,17,18], which could not only enhance our understanding of the basic principles underlying the action of dezocine but may also contribute to the further improvement of the quality of dezocine products.

Since critically ill surgical patients were unable to take oral medications [19], dezocine was generally used by intravenous injection. Accordingly, knowledge of the stability of dezocine injection is crucial to ensuring the safety of patients. Note that the packaging of marketed dezocine injection was ampoules with transparent glass. However, only a few studies reported the stability of dezocine [20], and there was a lack of research on the photostability of the dezocine injection. In the present study, we investigated two photodegradation products (named degradation products 1 and 2) in dezocine injection. First, the degradation products were separated and isolated via high-performance liquid chromatography (HPLC). Subsequently, the structure of these degradation products was characterized by a variety of spectral data (e.g., high-resolution MS, ^1^H NMR, ^13^C NMR, HSQC, and ^1^H-^1^H COSY). The possible mechanisms of their formation were proposed. Finally, the effect of light intensity and wavelength on the photodegradation of dezocine injection was investigated by monitoring the formation process of degradation products 1 and 2. The results of this study were beneficial for improving the quality control standards of dezocine injection and providing technical support for ensuring its safety.

## 2. Materials and Methods

### 2.1. Materials

Deionized water (18.2 Ω/cm) was prepared using a Milli-Q water purification system (Merck, Darmstadt, Germany). Normal and photodegraded samples of dezocine injection were provided by the Yangtze River Pharmaceutical Group. Acetonitrile (batch number: 194211) was purchased from Thermo Fisher Scientific, Inc. (Waltham, MA, USA). Sodium 1-heptanesulfonate (batch number: 20180323), ammonium formate (batch numbers: 20180323, 30011661), formic acid (batch number: 20190516), and phosphoric acid (batch number: 10015418) were obtained from Sinopharm Chemical Reagent Co., Ltd. (Shanghai, China). During the degradation study, the sample was first sealed with a transparent glass bottle and exposed to sunlight for 2 days. Then, the sample was stored in amber-colored glass. All reagents were of analytical grade or HPLC grade and used without further purification.

### 2.2. HPLC Conditions

The HPLC method was performed according to the standard [21]. A Shimadzu LC-30AD liquid chromatography (LC) system was used for measuring the chromatographic behavior and ultraviolet (UV) spectra of the degradation products. The analysis of the samples was performed on a Waters SunFire C18 column (250 mm × 4.6 mm, 5 μm). Mobile phase A was 5 mM sodium 1-heptanesulfonate (pH-adjusted to 3.5 with phosphoric acid)–acetonitrile (80:20). The mobile phase B was 5 mM sodium 1-heptanesulfonate (pH adjusted to 3.5 with phosphoric acid)–acetonitrile (20:80). The flow rate was 1.2 mL/min. The gradient elution program was 0 min, A: 100%, B: 0%; 35 min, A: 70%, B: 30%; 45–50 min, A: 30%; B: 70%; 51–60 min, A: 100%, B: 0%. The detector was a photodiode array (PDA), and the detection wavelength was 281 nm. The autosampler temperature was 4 °C. The injection volume of the sample was 20 μL. The normal and photodegraded samples of dezocine injection were diluted with mobile phase A to give an expected concentration of 1.5 mg/mL, respectively.

### 2.3. Preparative LC Conditions

For degradation product preparation, preparative LC (Waters 2545–Waters 2767) was performed on a Waters SunFire C18 Prep column (10 mm × 250 mm, 5 μm) by gradient elution (i.e., 0 min, A: 92%, B: 8%; 20 min, A: 90%, B: 10%; 45 min, A: 80%; B: 20%; 45–60 min, A: 92%, B: 8%). The mobile phase was 50 mM ammonium formate (pH adjusted to 3.5 with formic acid) (A) and acetonitrile (B) at a flow rate of 10.0 mL/min. The detector was a PDA, and the detection wavelength was 281 nm and 220 nm. The autosampler temperature was 4 °C. The injection volume of the sample was 1.5 mL.

### 2.4. LC-MS Conditions

A Dionex P680A two-dimensional LC system-tandem with mass spectrometry (MS) was used for the isolation and identification of the degradation products. The samples were separated on a Waters SunFire C18 (4.6 mm × 250 mm, 5 μm) column by gradient elution (A: 90–70% and B: 10–30% at 0–30 min) using 50 mM of ammonium formate (pH adjusted to 3.5 with formic acid) (A) and acetonitrile (B) as mobile phases. The flow rate was 0.9 mL/min. The detector was a PDA, and the detection wavelength was 281 nm. The autosampler temperature was 4 °C. The injection volume of the sample was 50 μL.

To identify the structure of the degradation products, a Thermo Fisher Scientific Q Exactive high-resolution MS was used to acquire the primary and secondary MS information of the degradation products. MS was performed using a heated electrospray ionization (ESI) source in the positive ion mode. The parameters for the heated ESI source were as follows: The capillary voltage was 3200 V (positive/negative ions). The sheath gas was 35 arb, and the auxiliary gas was 10 arb. The vaporization temperature was 300 °C. The scan range was *m*/*z* 50–2000, and the resolution was 75,000 Da. The scan mode included a full scan + automatic triggering of second-stage MS scanning. The collision mode was high-energy collision dissociation (HCD). The second-stage MS normalized collision energy was 25%.

### 2.5. NMR Spectroscopy Conditions

A Bruker AV-500 NMR spectrometer was used to identify the structure of the degradation products. A Bruker BioSpin Avance II 400 NMR spectrometer was used for quantitative analysis of the samples to acquire the correction factors of the degradation products. The ^1^H-NMR, ^13^C-NMR, ^1^H-^1^HCOSY, HSQC, and HMBC NMR spectra were recorded by using dimethyl sulfoxide-d6 (DMSO-d6) as the solvent with tetramethylsilane (TMS) as the internal reference.

### 2.6. Differential Scanning Calorimetry (DSC)

A TA DSC 2500 differential scanning calorimeter was used to analyze the enthalpy change in the degradation products under light irradiation. In total, 40 μL of the sample was placed into a DSC sample cup. An empty cup was used for reference. Then, both the sample cup and the reference cup were placed into the calorimeter. The temperature was set to 40 °C. After the sample was kept at 40 °C for 5 min, the shutter was opened. Subsequently, the sample was illuminated for 60 min. Finally, the DSC data were recorded.

## 3. Results and Discussion

### 3.1. Isolation of the Photodegradation Products in the Dezocine Injection

**Figure 1:**
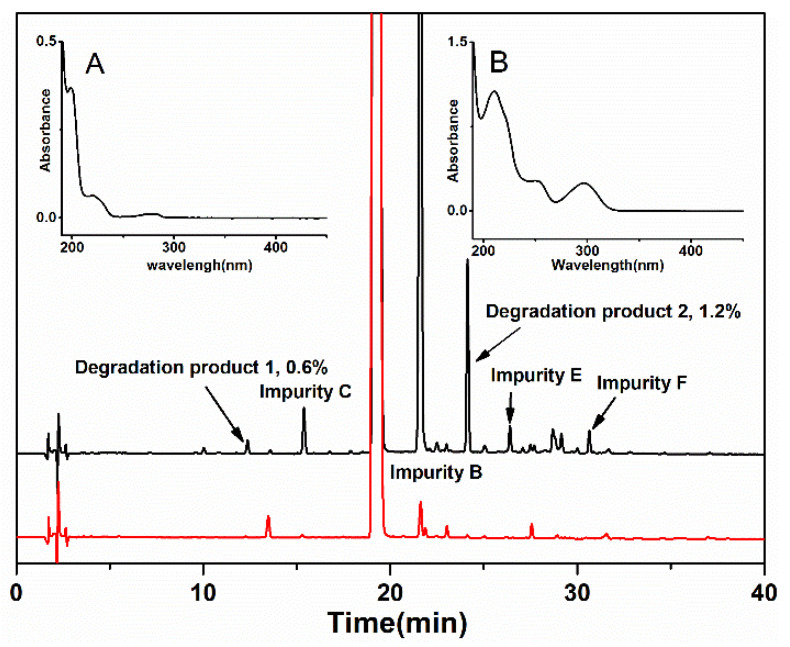
Chromatograms of the photodegraded sample (black line) and normal sample (red line) were obtained from the sodium 1-heptanesulfonate chromatographic system. Inset: UV spectra of degradation product 1 (**A**) and degradation product 2 (**B**) extracted from the sodium 1-heptanesulfonate chromatographic system.

Firstly, the chromatograms of the normal and photodegraded samples of dezocine injection are shown in Figure 1. Degradation product 1 and degradation product 2 could be identified through the comparison of chromatograms of the normal and photodegraded samples. Degradation product 1 and degradation product 2 were effectively separated at the retention times of 12.37 min and 24.13 min, respectively. The normalized area of degradation product 1 and degradation product 2 in the photodegraded sample was 0.6% and 1.2%, respectively. The results indicated that the content of these two degradation products was relatively high in the photodegraded sample of dezocine injection and was higher than the thresholds for degradation products in new drug products in the ICH Q3B(R) guideline [22]. Moreover, since the other impurities were well studied (Figure 1 and Table S1), degradation product 1 and degradation product 2, as the unknown impurities generated during the photodegradation process of the dezocine injection, were chosen for the following study. The UV spectra of degradation product 1 and degradation product 2 extracted from the sodium 1-heptanesulfonate chromatographic system are shown in the inset of Figure 1.

### 3.2. Structure Identification of the Photodegradation Products in the Dezocine Injection

**Figure 2:**
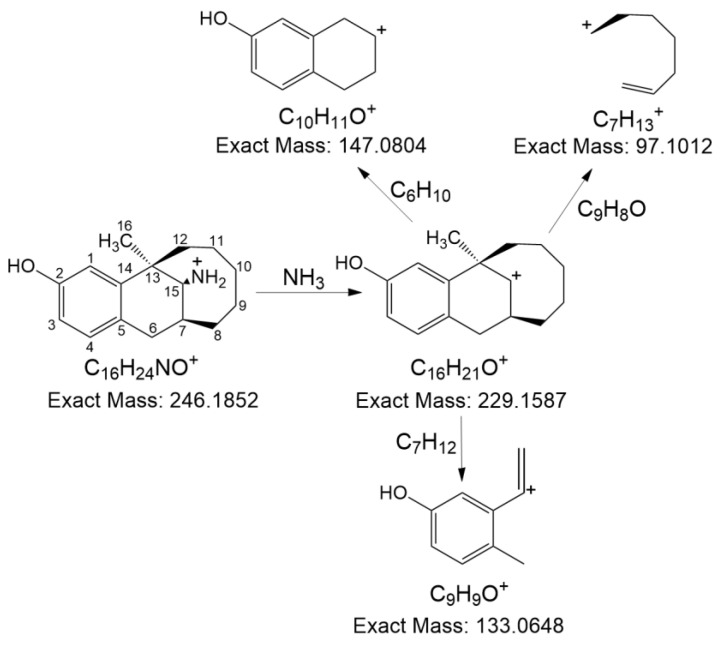
The MS fragmentation pathway of dezocine.

The sample of degradation products 1 and 2 was prepared using the preparative LC method. After freeze-drying and redissolving in deionized water five times, approximately 40 mg of degradation product 1 and 70 mg of degradation product 2 were obtained. Firstly, the structure of dezocine was confirmed by high-resolution MS. As shown in Figure S1, dezocine provided a protonated molecule [M+H]^+^ at *m*/*z* 246.18433, indicating the elemental composition was C_16_H_24_NO^+^. Figure 2 shows the MS fragmentation pathway of dezocine. Further confirmation was achieved through the ^1^H NMR, ^13^C NMR, ^1^H–^1^HCOSY, HSQC, and HMBC spectra (Figures S2–S6). The ^1^H NMR and ^13^C NMR data of dezocine are listed in Table 1. Note that the two degradation products (i.e., degradation product 1 and degradation product 2) were only found in dezocine injection instead of the active pharmaceutical ingredient (API) (i.e., dezocine).

**Figure 3:**
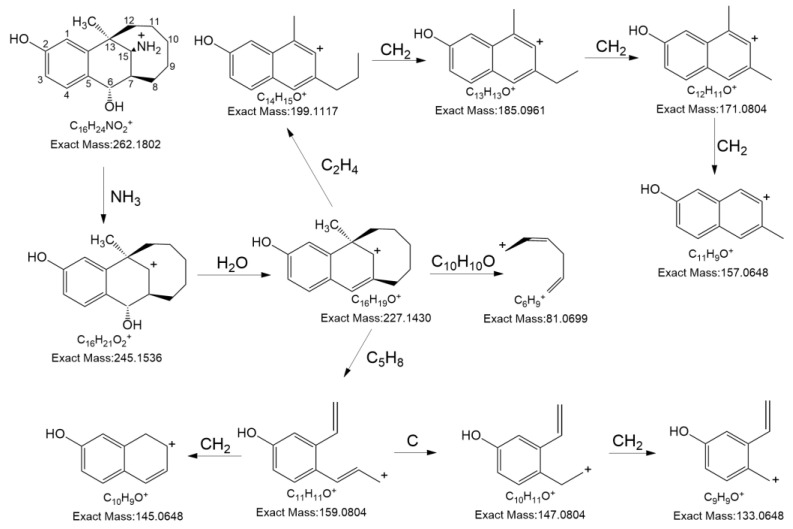
The MS fragmentation pathway of degradation product 1.

Subsequently, the structure of degradation product 1 was elucidated and validated based on the data acquired on the high-resolution MS and NMR spectroscopy. The results of high-resolution MS indicated that the protonated molecule [M+H]^+^ of degradation product 1 was 262.17896 (Figure S7), which was 16 *m*/*z* units higher than the protonated molecule [M+H]^+^ of the dezocine (i.e., 246.18433). Dezocine was structurally similar to morphine. It is reported in the literature that both benzylic and allylic positions exist in morphine molecules, which can undergo the oxidation reaction to produce two types of degradation products [23]. Accordingly, it was speculated that degradation product 1 was the oxidation product of dezocine. Figure 3 shows the structure and MS fragmentation pathway of degradation product 1.

NMR spectroscopy played a vital role in structural confirmation, which enabled the underlying mechanisms of the degradation or formation process of impurities to be revealed [9]. The NMR analysis results provided further support for the correctness of the speculated degradation product 1 structure. In particular, the chemical shift of C-6 in degradation product 1 (i.e., 72.90 ppm) was shifted downfield in comparison to that in dezocine (35.36 ppm), indicating that the hydroxyl group was attached to C-6. Table 1 shows the 1H-NMR and ^13^C-NMR data of degradation product 1. The ^1^H NMR, ^13^C NMR, COSY, HSQC, and HMBC spectra were presented in Figures S8–S12. Since degradation product 1 was unstable during the preparation process, the amount of degradation product 1 was small. Based on the results from high-resolution mass spectrometry (Figure S7) and NMR spectrometry (Table 1), the key sites in the structure of degradation product 1 were confirmed. As a result, we did not use the DEPT 135 spectrum to confirm the conversion of -CH2 in dezocine into -CH in degradation product 1 at the C-6 position.

**Figure 4:**
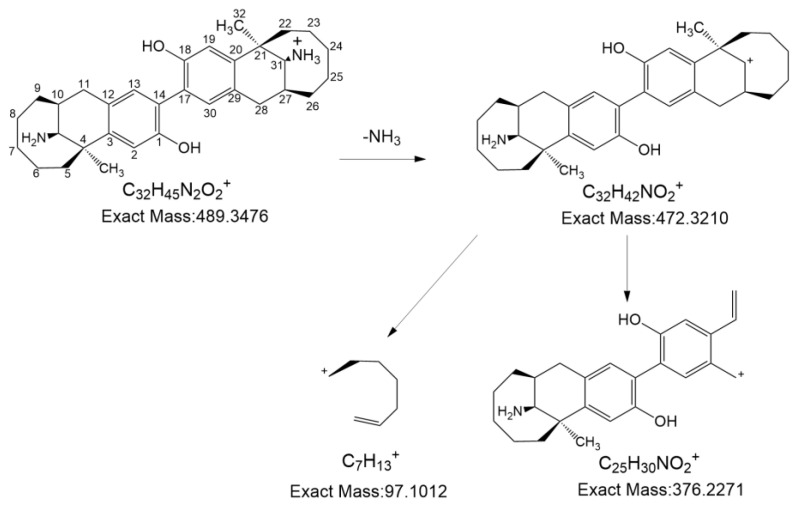
The MS fragmentation pathway of degradation product 2.

On the other hand, the results of HRMS indicated that degradation product 2 had a protonated molecule [M+H]^+^ of 489.34546 (Figure S13), and dezocine had a protonated molecule [M+H]^+^ of 246.18433. Since dezocine contains easy substitution sites (i.e., the ortho-position of phenol hydroxyl structure), it is reported in the literature that morphine with a similar structure could undergo autoxidation to produce carbon–carbon (C-C)-coupled dimers [24]. Therefore, it was speculated that degradation product 2 may be a dimer formed by the loss of two hydrogen atoms from the dezocine. Figure 4 shows the possible structure and MS fragmentation pathway of degradation product 2. The NMR analysis results provided further support for the correctness of the speculated degradation product 2 structure. Analysis of the ^1^H-NMR data suggests the signal assignments to the following protons on the benzene ring of the dezocine: H1 (δ = 6.60 ppm, 6.59 ppm; doublet), H4 (δ = 6.38 ppm, 6.38 ppm; doublet), and H3 (δ = 6.27–6.28 ppm; quartet). The ^1^H NMR, ^13^C NMR, COSY, HSQC, and HMBC spectra of degradation product 2 are presented in the Supplementary Material (Figures S14–S18). Table 1 shows the ^1^H-NMR and ^13^C NMR data of degradation product 2. For degradation product 2, the ^1^H NMR data indicated that it retained the signals of two hydrogens (δ = 6.92 ppm, 6.74 ppm) on the benzene ring. The COSY spectrum revealed an absence of cross-peaks between the two peaks, suggesting that the peaks corresponded to the two hydrogens in the para-position of the benzene ring. By comparing the ^1^H NMR spectra of degradation product 2 and dezocine, it was also revealed that the two hydrogens in the para-position of the benzene ring of dezocine were retained in degradation product 2. An analysis of the HMBC spectrum indicated that the peak at δ = 115.40 ppm in the carbon spectrum belonged to C2 and C19, and the peak at δ = 131.66 belonged to C13 and C30. In addition, the peak at δ = 153.53 ppm belonged to C1 and C18, each with an attached hydroxyl group. Therefore, degradation product 2 was determined as a dimer of dezocine. These results provide further confirmation of the correctness of the speculated structure of degradation product 2 based on MS. We also studied the degradation of dezocine injections triggered by different conditions (i.e., acid, alkali, heat, and oxidative degradation). The results showed that Impurity B was generated during the degradation process of dezocine injection under alkali conditions or acid conditions. The three conditions (i.e., acid, alkali, and heat degradation) had no effect on the formation of degradation product 1 and degradation product 2. We found that a small amount of degradation product 1 was generated under the oxidative degradation condition.

### 3.3. Mechanism of the Formation Process of Degradation Products

**Figure 5:**
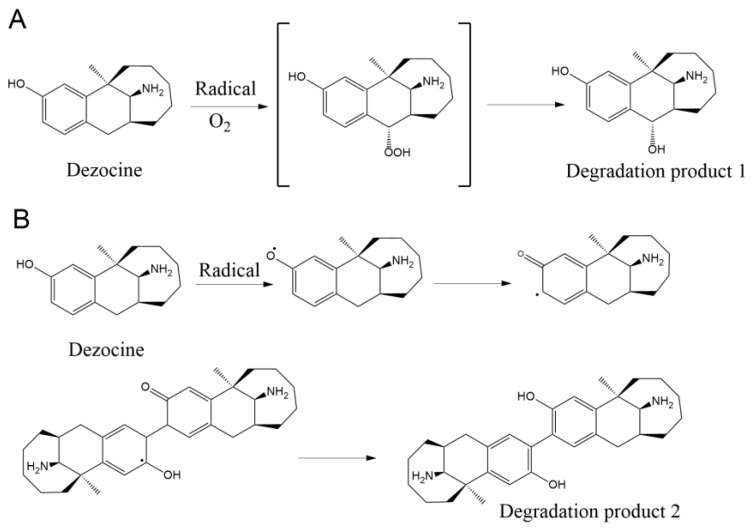
The photodegradation pathways of dezocine including the formation process of degradation product 1 (**A**) and degradation product 2 (**B**).

To better understand the photodegradation properties of dezocine injection, it is important to evaluate the formation process of degradation products 1 and 2. As shown in Figure 5, the photodegradation pathway of dezocine was estimated based on the chemical structure of degradation products 1 and 2. Note that since the structure of dezocine is similar to some of the structures of morphine, the degradation mechanism of dezocine refers to the degradation process of morphine [23,24,25]. Briefly, for degradation product 1, position 11 of dezocine could be oxidized by an oxygen radical, which can form in the injection under light irradiation. Thus, the hydroxyl group was formed in position 11 of dezocine through a hydroperoxyl intermediate. On the other hand, the phenol structure in dezocine could be easily oxidized to form the phenoxyl radical. Subsequently, the radical could be transferred to position 2 of the benzene ring by resonance. Finally, after the radical attacked another dezocine molecule to form a dimer-free radical, degradation product 2 was generated by the autoxidation of the dimer-free radical (Figure 5B). In conclusion, based on the established degradation mechanism of morphine, we speculated on the photodegradation mechanism of dezocine injection. Furthermore, we found that only a small amount of degradation product 1 was generated under the oxidative degradation condition. Degradation product 1 and degradation product 2 were not found in the other degradation processes (i.e., acid, alkali, and heat degradation). The results fully demonstrate that our speculated mechanism of the two degradation products was correct, and no other mechanism was involved.

### 3.4. Effect of Light Intensity and Wavelength on the Photodegradation of Dezocine Injection

**Figure 6:**
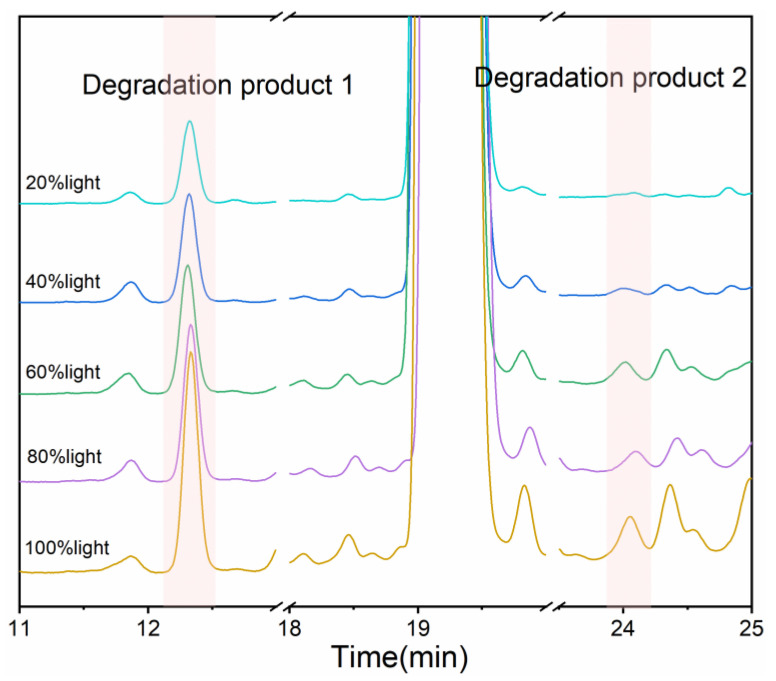
Chromatograms of dezocine injection for 24 h under different illumination intensity.

**Figure 7:**
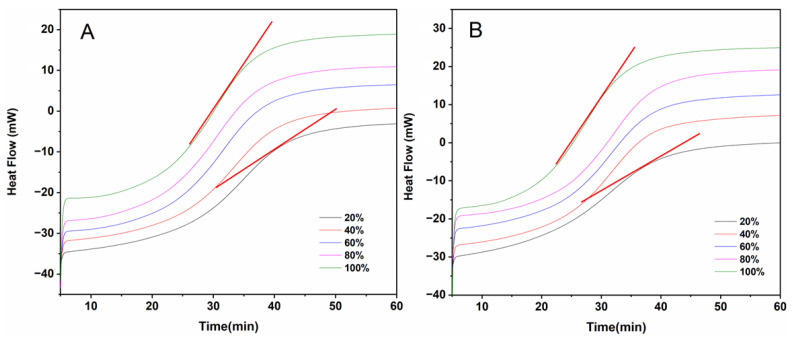
DSC curve of dezocine injection under ultraviolet light (**A**) and visible light (**B**) with different illumination intensities.

In order to investigate the effect of light intensity and wavelength on the photodegradation of dezocine injection, we monitored the formation process of degradation products 1 and 2 using the HPLC with a PDA detector and the DSC with a light source. The dezocine injection was illuminated for 60 min with full-spectrum light at different light intensities (i.e., 100%, 80%, 60%, 40%, and 20%). After the light irradiation, we immediately detected the amount of the degradation products and the dezocine to evaluate the formation of degradation products and degradation of drugs. Interestingly, few degradation products were produced, and the dezocine almost did not degrade. After 24 h following the exposure of the sample, degradation products 1 and 2 appeared in the dezocine injection. The amount of degradation products 1 and 2 increased with the increase in light intensity. Briefly, with the increase in light intensity from 20% to 100%, the peak area percentage of degradation product 1 increased from 0.157% to 0.456%, and the peak area percentage of dezocine decreased from 99.045% to 90.733% (Figure 6 and Table 2). Degradation product 2 appeared in small amounts only at high-light intensity. In addition, the effect of light wavelength on the photodegradation of dezocine injection was further evaluated using a 320–390 nm (i.e., ultraviolet light) filter (Lumen Dynamics Group Inc., Mississauga, ON, Canada) and 400–500 nm (i.e., visible light) filter (Lumen Dynamics Group Inc., Mississauga, ON, Canada), respectively. The slope of the DSC curve could reflect the rate of the photodegradation reaction [26,27]. The slope of the DSC curve under ultraviolet light was larger than that under visible light (Figure 7), suggesting that the dezocine injection was more sensitive to ultraviolet light than visible light.

## 4. Conclusions

In this study, we identified the chemical structure of two photodegradation products (i.e., degradation product 1 and degradation product 2) in dezocine injections. Degradation product 1 was confirmed as the oxidation product of dezocine. Degradation product 2 was confirmed as the coupled dimer of dezocine. Under light irradiation, the degradation efficiency of dezocine increased with the increase in light intensity. Importantly, we found that the formation of degradation product 1 and degradation product 2 required not only light to trigger the reaction but also a longer reaction time. Furthermore, ultraviolet light with higher energy could cause the degradation of dezocine injection easily in comparison to visible light. These results are important for the quality control, standard enhancement, and product optimization of dezocine injection. It was also crucial to clarify the impact of photo exposure on both the quality and quantity of photosensitive pharmaceuticals to ensure their safe use. It was important to control the levels of both degradation products found in the injection formulation of dezocine.

## Figures and Tables

**Table 1:** ^1^H NMR and ^13^C date (δ) for photodegraded API, degradation product 1, and degradation product 2 in DMSO–*d*_6._

API (Dezocine)				Degradation Product 1				Degradation Product 2			
Chemical Shift (ppm)	^1^H	Chemical Shift (ppm)	^13^C	Chemical Shift (ppm)	^1^H	Chemical Shift (ppm)	^13^C	Chemical Shift (ppm)	^1^H	Chemical Shift (ppm)	^13^C
6.378 (d, J = 2.54 Hz, 1H)	C1-H	114.05	C1	6.61 (d, J = 2.3 Hz, 1H)	C1-H	114.05	C1	6.92 (s, 2H)	C13-H, C30-H	152.60	C1, C18
		155.45	C2			157.48	C2	6.74 (s, 2H)	C2-H, C19-H	141.59	C3, C20
6.274 (dd, J = 2.54, 8.22, 1H)	C3-H	113.65	C3	6.57 (dd, J = 8.1, 2.2 Hz, 1H)	C3-H	114.38	C3	3.23–3.18 (m, 2H)	C15-H, C31-H	130.74	C13, C30
6.593 (d, J = 8.22 Hz, 1H)	C4-H	129.47	C4	7.02 (d, J = 8.2 Hz, 1H)	C4-H	132.35	C4	3.04 (dd, *J* = 16.1, 6.6 Hz, 2H)	C11-H, C28-H	125.70	C12, C29
		126.54	C5			129.95	C5	2.59 (d, *J* = 16.5 Hz, 2H)	C11-H, C28-H	124.29	C14, C17
2.267, 2.730 (d, dd, J = 16.20, 7.20, 1H)	C6-H	35.36	C6	4.30 (s, 1H)	C6-H	72.90	C6	2.23 (s, 2H)	C10-H, C27-H	114.47	C2, C19
1.845 (m, 1H)	C7-H	37.45	C7	2.12–2.03 (m, 1H)	C7-H	45.94	C7	1.99–1.93 (m, 2H)	C5-H, C22-H	58.09	C15, C31
1.516, 1.304 (dt, t, 1H)	C8-H	29.60	C8	1.68–1.64 (m, 1H)	C8-H			1.77–1.71 (m, 2H)	C9-H, C26-H	40.06	C4, C21
0.578, 1.282 (dt, t, 1H)	C9-H	22.91	C9	1.43–1.34 (m, 3H)	C8-H, C10-H			1.66–1.64 (m, 2H)	C9-H, C26-H	36.08	C5, C22
0.852, 1.207 (dt, t, 1H)	C10-H	29.86	C10	1.09–0.97 (m, 2H)	C9-H			1.62–1.59 (m, 2H)	C5-H, C22-H	35.32	C10, C27
0.512, 1.176 (dt, t, 1H)	C11-H	26.13	C11	0.71–0.54 (m, 2H)	C11-H			1.50–1.44 (m, 6H)	C6-H, C7-H, C8-H, C23-H, C24-H, C25-H	34.58	C16, C32
1.263, 1.805 (dt, t, 1H)	C12-H	36.59	C12	2.03–1.92, 1.53–1.47 (m, 1H)	C12-H	36.99	C12	1.34 (s, 6H)	C16-H, C32-H	34.19	C11, C28
		40.49	C13			40.98	C13	1.20–1.12 (m, 2H)	C7-H, C24-H	28.98	C9, C26
		144.93	C14			145.88	C14	0.88–0.85 (m, 2H)	C8-H, C25-H		
2.742 (d, J = 4.90Hz, 1H)	C15-H	58.17	C15	3.40 (d, J = 4.6 Hz, 1H)	C15-H	53.33	C15	0.84–0.78 (m, 2H)	C6-H, C23-H		
1.022 (s, 1H)	C16-H	35.41	C16	1.27 (s, 3H)	C16-H	35.41	C16				C16

**Table 2:** Chromatographic data analysis of different target analytes in dezocine injection for 24 h under different illumination intensities.

Target Analyte	Light Treatment	Retention Time	Area	Area%
Dezocine	20%	19.091	15,078,851	99.045
	40%	19.104	14,942,159	97.770
	60%	19.078	14,883,555	96.211
	80%	19.150	12,637,760	94.425
	100%	19.112	12,308,597	90.733
Degradation product 1	20%	12.324	23,923	0.157
	40%	12.319	31,389	0.205
	60%	12.308	38,302	0.248
	80%	12.333	43,686	0.326
	100%	12.334	61,803	0.456
Degradation product 2	20%	24.085	887	0.006
	40%	24.000	2623	0.019
	60%	24.017	6748	0.049
	80%	24.096	7248	0.053
	100%	24.053	14,322	0.106

## Data Availability

Data are available from the authors.

## Supplementary Materials

The following supporting information can be downloaded at https://www.mdpi.com/article/10.3390/pharmaceutics16070858/s1, Figure S1: Mass spectra of dezocine; Figure S2: 1H NMR spectra of dezocine; Figure S3: 13C NMR spectrum of dezocine; Figure S4: COSY NMR spectrum of dezocine; Figure S5: HSQC NMR spectrum of dezocine; Figure S6: HMBC NMR spectrum of dezocine; Figure S7: Mass spectra of degradation product 1; Figure S8: 1H NMR spectrum of degradation product 1; Figure S9: 13C NMR spectrum of degradation product 1; Figure S10: COSY NMR spectrum of degradation product 1; Figure S11: HSQC NMR spectrum of degradation product 1; Figure S12: HMBC NMR spectrum of degradation product 1; Figure S13: Mass spectrum of degradation product 2; Figure S14: 1H NMR spectrum of degradation product 2; Figure S15: 13C NMR spectrum of degradation product 2; Figure S16: COSY NMR spectrum of degradation product 2; Figure S17: HSQC NMR spectrum of degradation product 2; Figure S18: HMBC NMR spectrum of degradation product 2; Table S1: The structure of the known impurities in Figure 1.
