# Supplementary material for: Influence of Light Irradiation on the Degradation of Dezocine in Injections
Source: Pharmaceutics. 2024 Jun 25;16(7):858. doi: 10.3390/pharmaceutics16070858 (PMC11279387; doi:10.3390/pharmaceutics16070858)
Supplement: Supplementary file 1 [file pharmaceutics-16-00858-s001.zip › pharmaceutics-3044712-supplementary.pdf]

Supplementary Material

Influence of Light Irradiation on the Degradation of Dezocine in Injection

Li Zhu [1,†], Xu Teng [2,†,*], Yu Duan [1,†], Xia Zhang [1], Jingxin Xie [1], Mingzhe Xu [1], and Lihui Yin [1,*]

[1] Key Laboratory for Quality Research and Evaluation of Chemical Drugs, National Institutes for Food and Drug Control, Beijing 100029, China; zhuli@nifdc.org.cn (L.Z.); yduane@163.com (Y.D.); s791657998@126.com (X.Z.); xjejx@nifdc.org.cn (J.X.); xumzhe@nifdc.org.cn (M.X.);

[2] Department of Laboratory Medicine, Affiliated Qingyuan Hospital of Guangzhou Medical University, Qingyuan People's Hospital, 511518, Qingyuan, Guangdong, China;

* Correspondence: xu0601@163.com (X.T.); yinlihui@vip.163.com (L.Y.);

† These authors contributed equally to this work.

Contents

Figure S1. Mass spectrum of dezocine

Figure S2. ^{1}H NMR spectra of dezocine

Figure S3. ^{13}C NMR spectrum of dezocine

Figure S4. COSY NMR spectrum of dezocine

Figure S5. HSQC NMR spectrum of dezocine

Figure S6. HMBC NMR spectrum of dezocine

Figure S7. Mass spectrum of degradation product 1

Figure S8. ^{1}H NMR spectrum of degradation product 1

Figure S9. ^{13}C NMR spectrum of degradation product 1

Figure S10. COSY NMR spectrum of degradation product 1

Figure S11. HSQC NMR spectrum of degradation product 1

Figure S12. HMBC NMR spectrum of degradation product 1

Figure S13. Mass spectrum of degradation product 2

Figure S14. ^{1}H NMR spectrum of degradation product 2

Figure S15. ^{13}C NMR spectrum of degradation product 2

Figure S16. COSY NMR spectrum of degradation product 2

Figure S17. HSQC NMR spectrum of degradation product 2

Figure S18. HMBC NMR spectrum of degradation product 2

Table S1. The structure of the known impurities in Figure 1

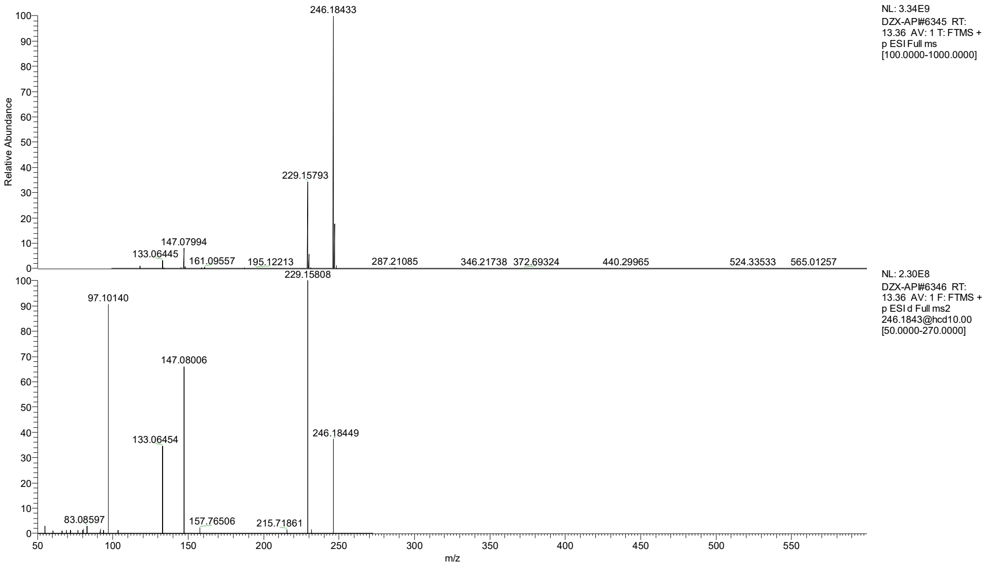

Figure S1. Mass spectrum of dezocine

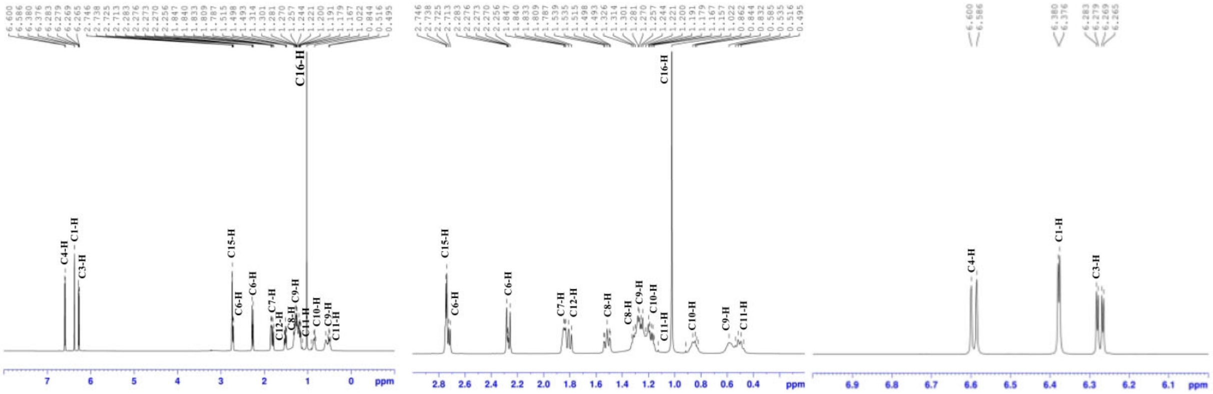

Figure S2. ^{1}H NMR spectra of dezocine

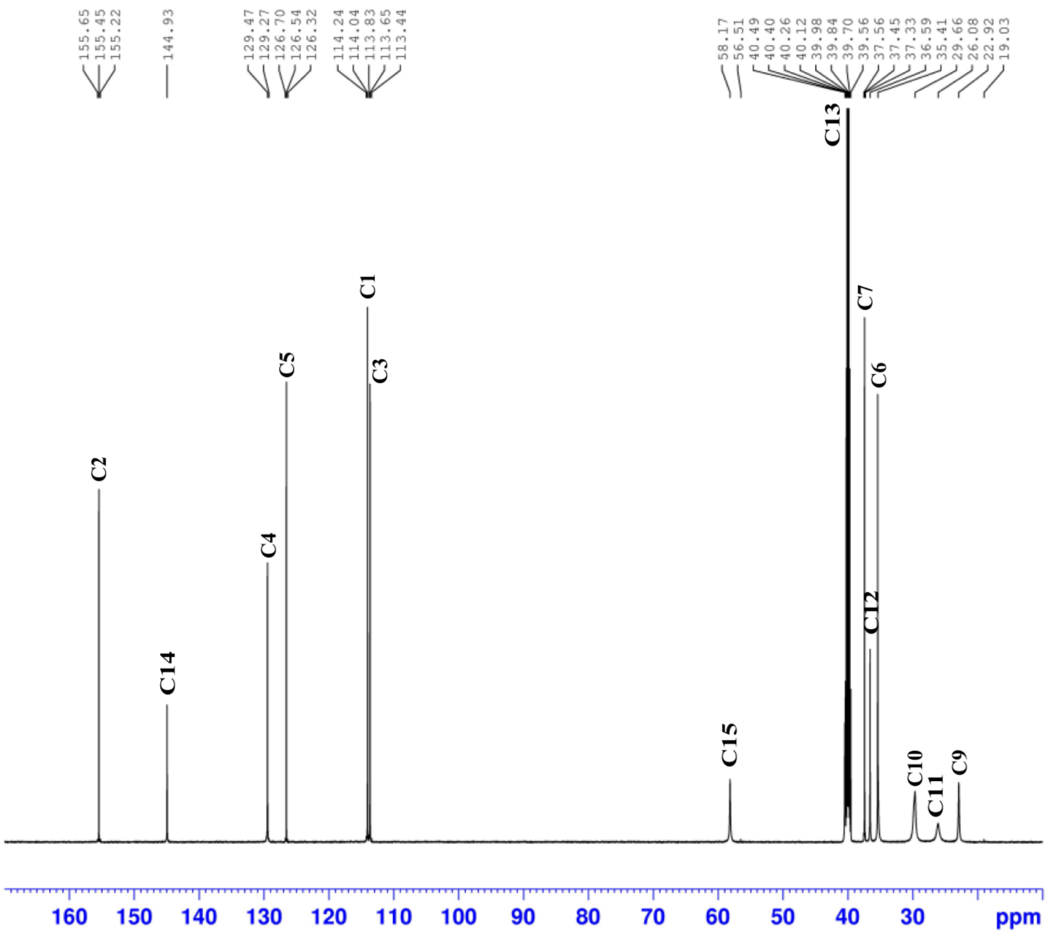

Figure S3. ^{13}C NMR spectrum of dezocine

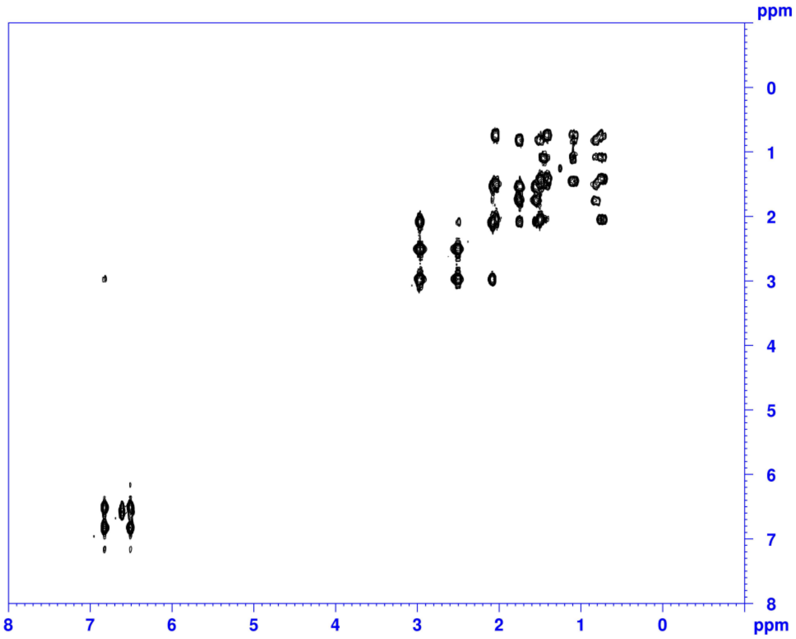

Figure S4. ^{1}H–^{1}HCOSY NMR spectrum of dezocine

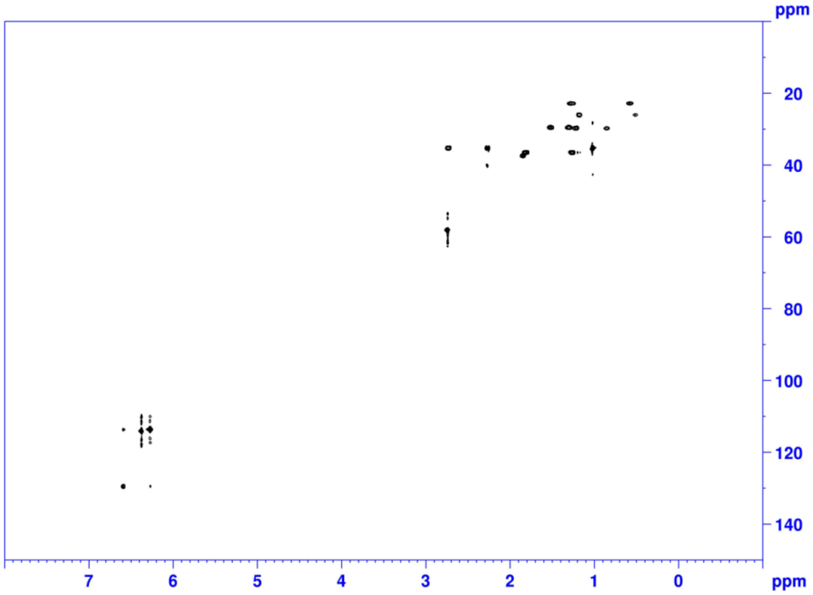

Figure S5. HSQC NMR spectrum of dezocine

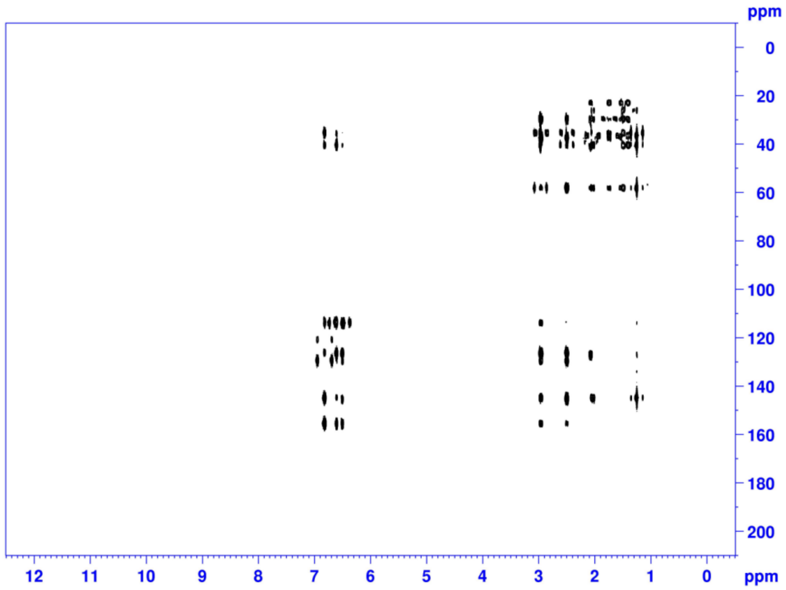

Figure S6. HMBC NMR spectrum of dezocine

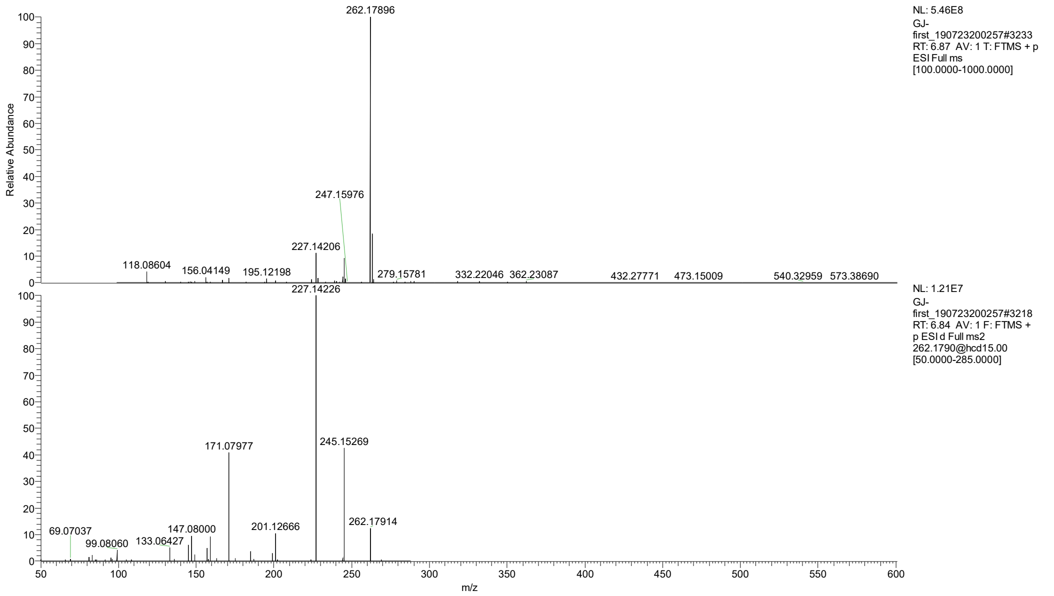

Figure S7. Mass spectrum of degradation product 1

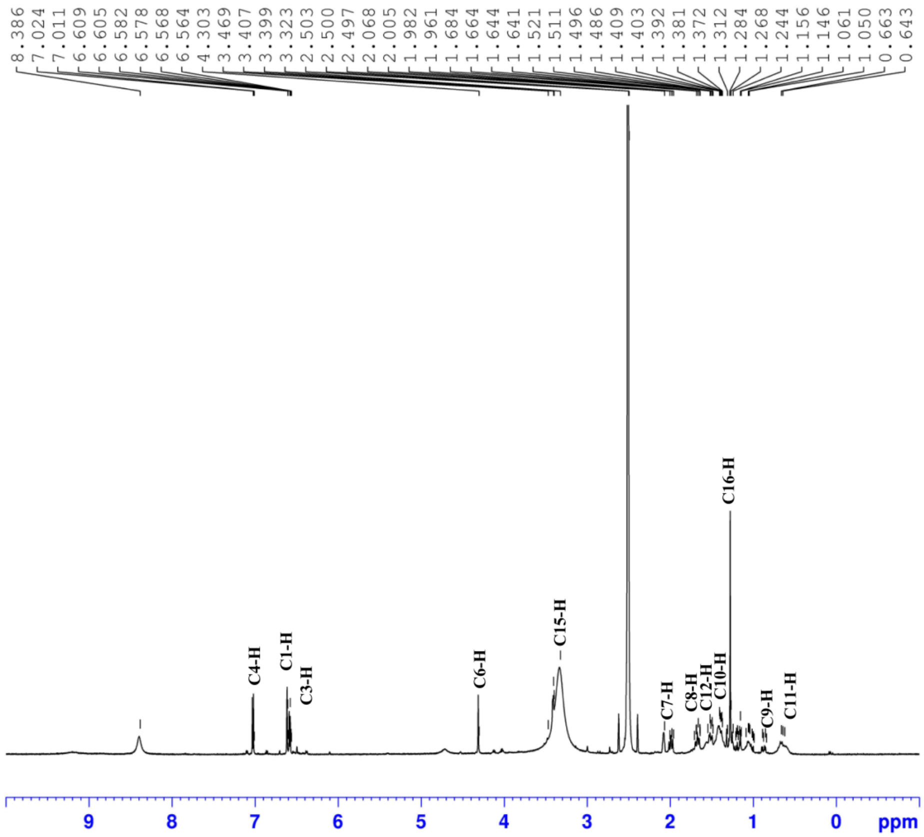

Figure S8. ^{1}H NMR spectrum of degradation product 1

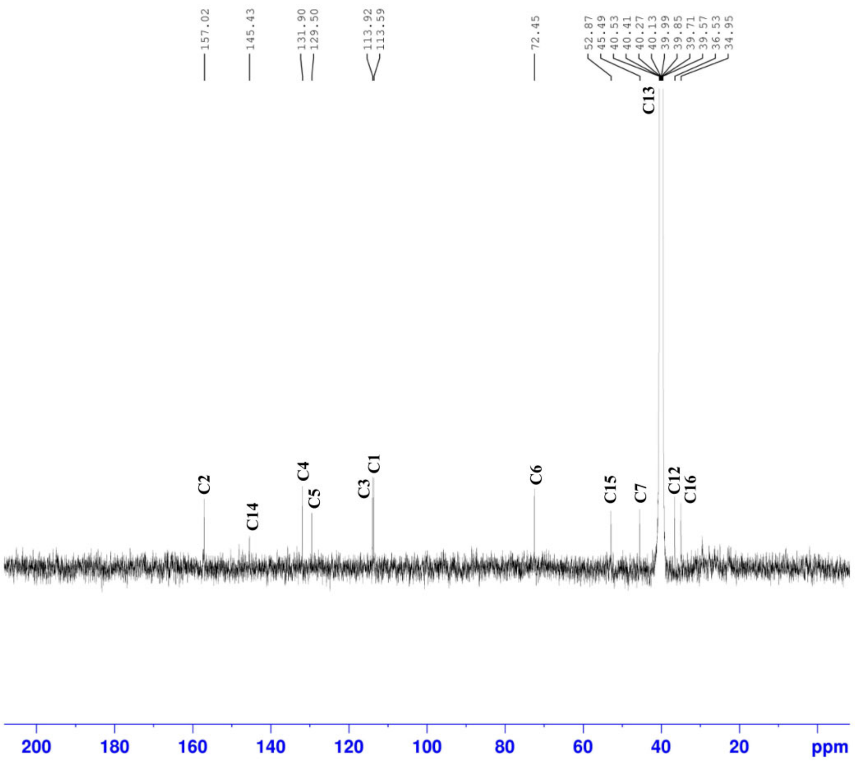

Figure S9. ^{13}C NMR spectrum of degradation product 1

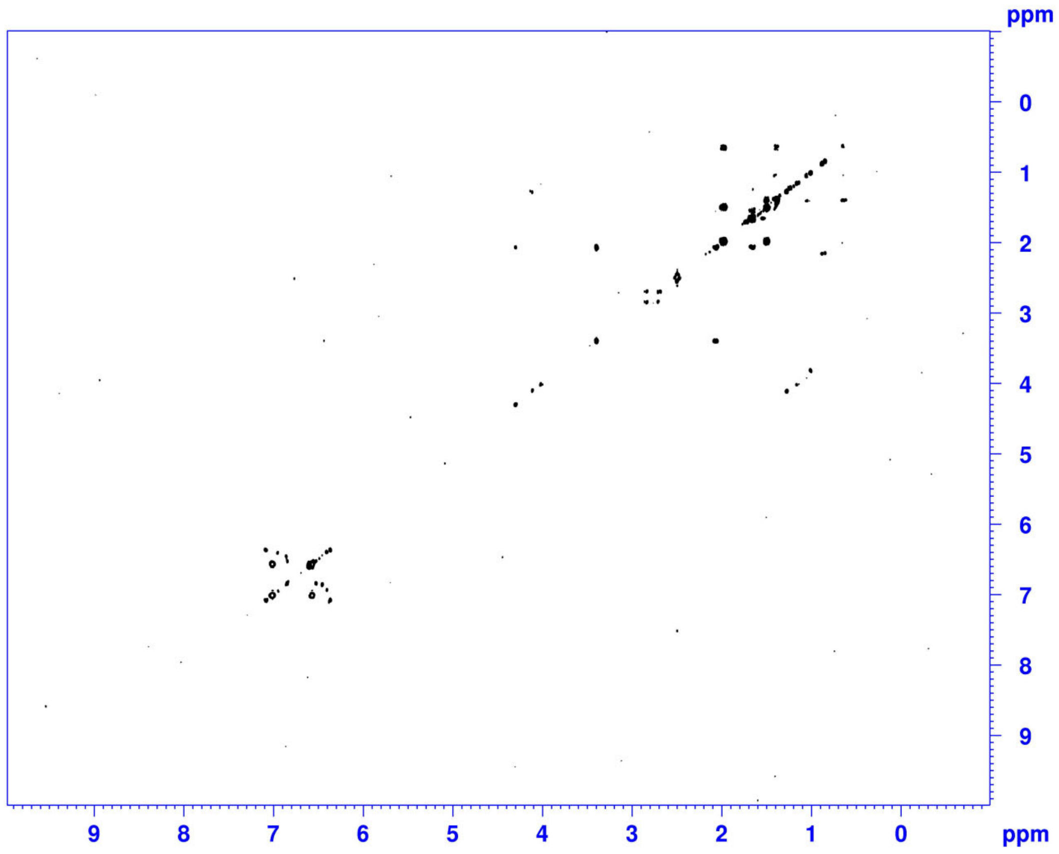

Figure S10. COSY NMR spectrum of degradation product 1

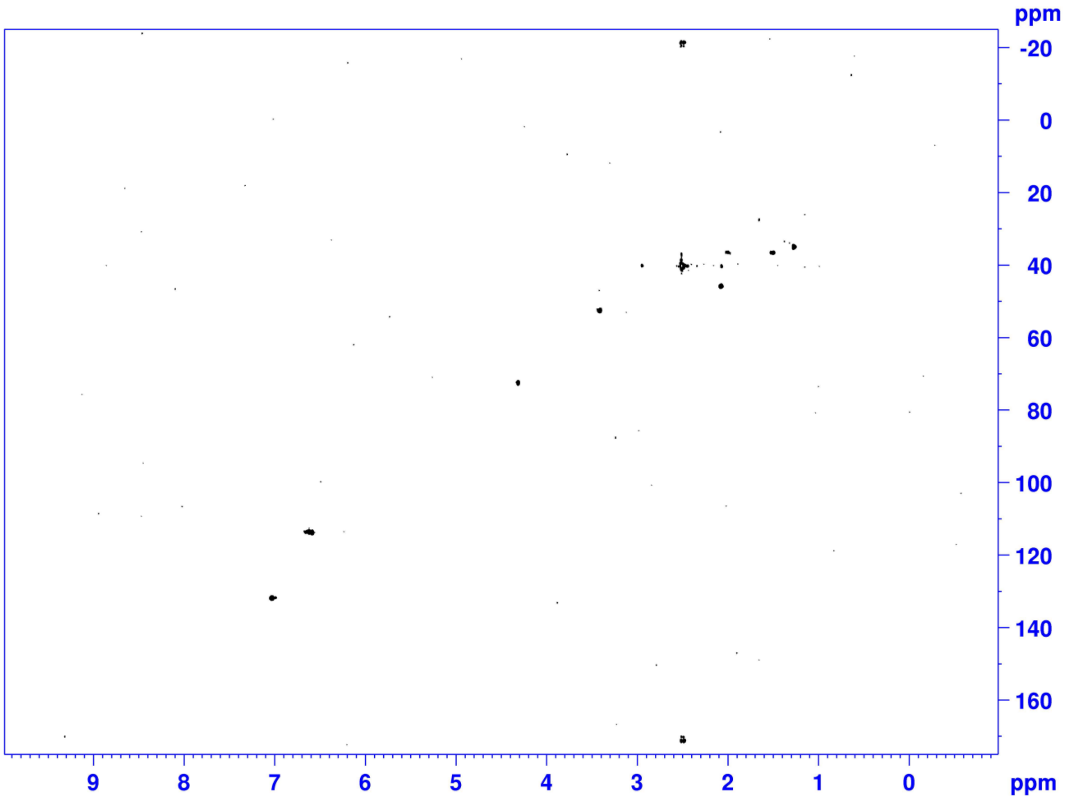

Figure S11. HSQC NMR spectrum of degradation product 1

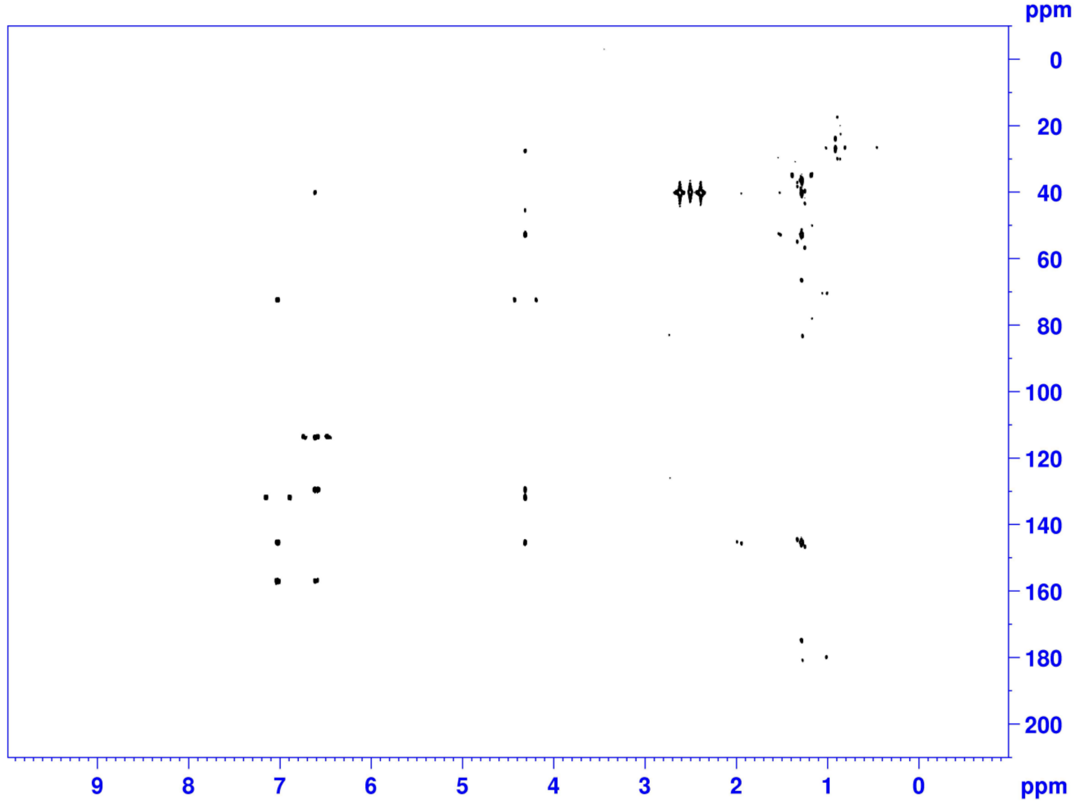

Figure S12. HMBC NMR spectrum of degradation product 1

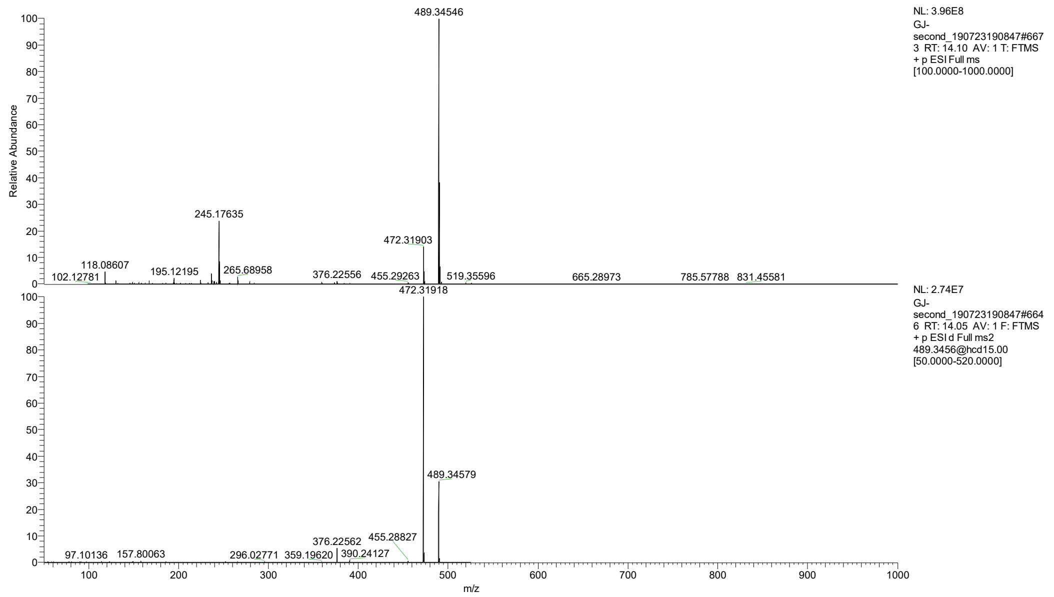

Figure S13. Mass spectrum of degradation product 2

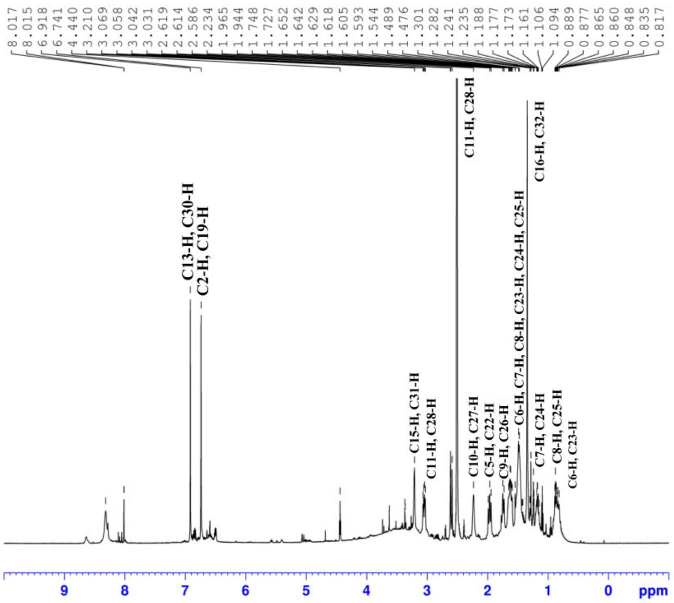

Figure S14. ^{1}H NMR spectrum of degradation product 2

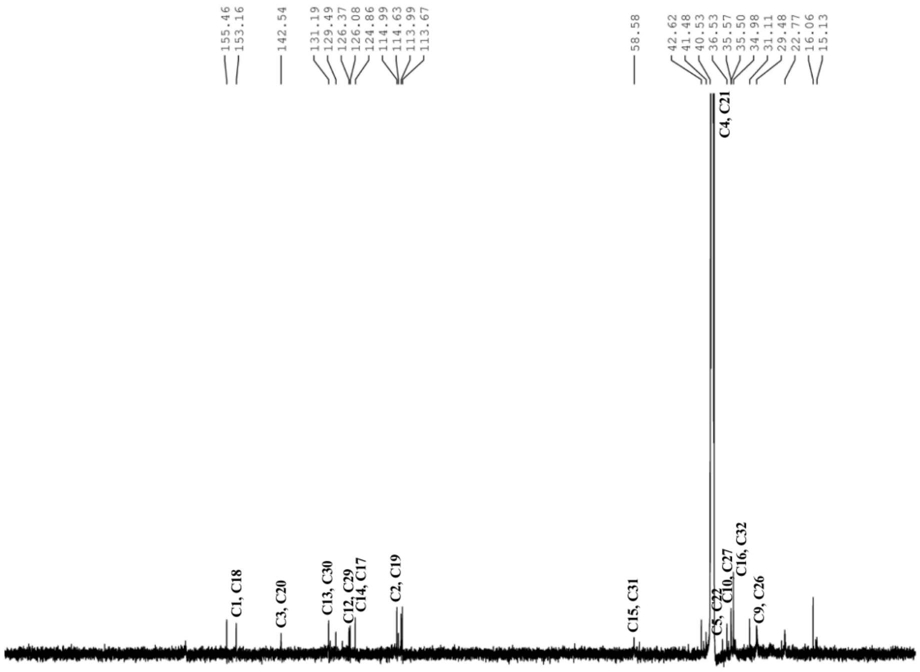

Figure S15. ^{13}C NMR spectrum of degradation product 2

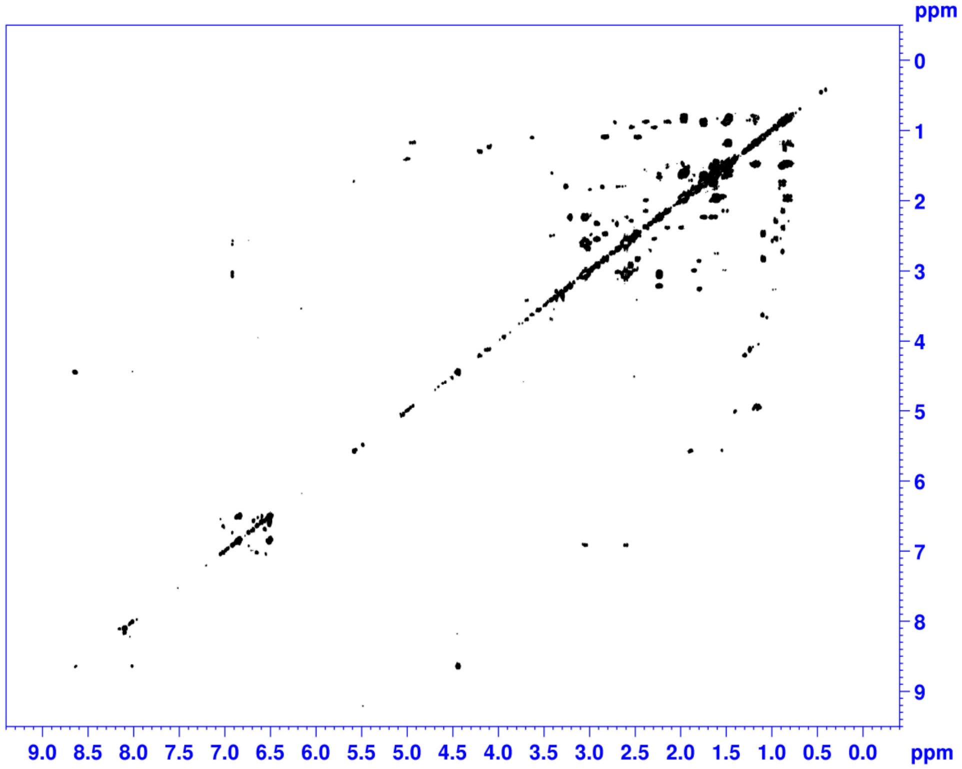

Figure S16. COSY NMR spectrum of degradation product 2

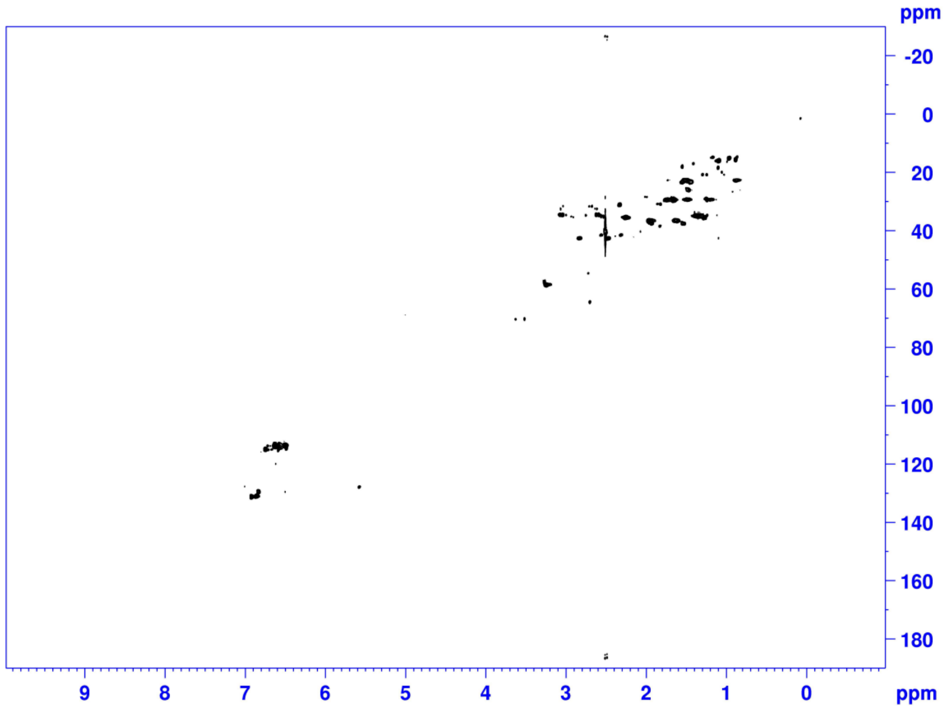

Figure S17. HSQC NMR spectrum of degradation product 2

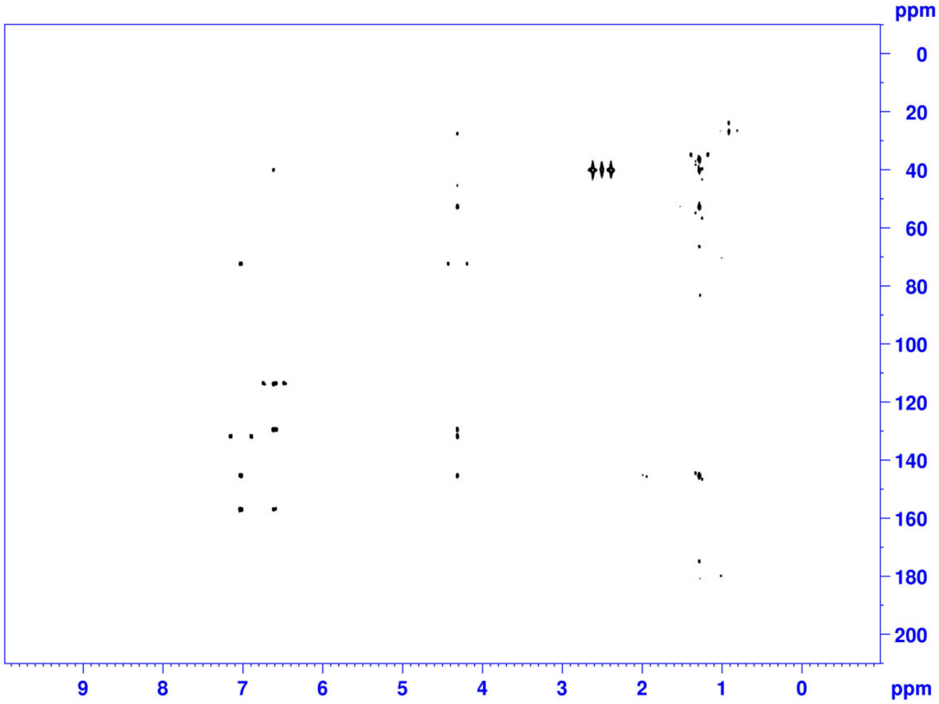

Figure S18. HMBC NMR spectrum of degradation product 2

Table S1. The structure of the known impurities in Figure 1

Name	Structure
Impurity B	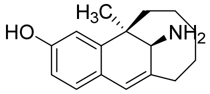
Impurity C	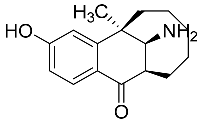
Impurity E	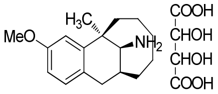
Impurity F	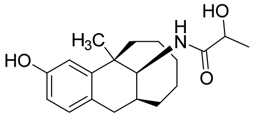
